# The clinical use of Kampo medicines (traditional Japanese herbal treatments) for controlling cancer patients’ symptoms in Japan: a national cross-sectional survey

**DOI:** 10.1186/1472-6882-12-222

**Published:** 2012-11-20

**Authors:** Satoru Iwase, Takuhiro Yamaguchi, Tempei Miyaji, Kiyoshi Terawaki, Akio Inui, Yasuhito Uezono

**Affiliations:** 1 Department of Palliative Medicine, The University of Tokyo Hospital, 7-3-1 Hongo, Bunkyo-ku, Tokyo, 113-0033, Japan; 2 Division of Biostatistics, Tohoku University Graduate School of Medicine, 1-1 Seiryo-machi, Aoba-ku, Sendai, Miyagi, 980-8574, Japan; 3 Interfaculty Initiative in Information Studies, The University of Tokyo, 7-3-1 Hongo, Bunkyo-ku, Tokyo, 113-0033, Japan; 4 Division of Cancer Pathophysiology, National Cancer Center Research Institute, 5-1-1 Tsukiji, Chuo-ku, Tokyo, 104-0045, Japan; 5 Department of Psychosomatic Internal Medicine, Kagoshima University Graduate School of Medical and Dental Sciences, 8-35-1 Sakuragaoka, Kagoshima, 890-8520, Japan

**Keywords:** Kampo, Kampo medicine, Palliative care, Symptom management, Survey

## Abstract

**Background:**

Kampo medicines are traditional Japanese medicines produced from medicinal plants and herbs. Even though the efficacy of Kampo medicines for controlling cancer-related symptoms is being reported, their actual nationwide clinical use has not been comprehensively investigated. We aimed to investigate physicians’ recognition of Kampo medicines and their clinical use for cancer patients in the field of palliative care.

**Methods:**

A cross-sectional self-administered anonymous questionnaire was distributed to 549 physicians working in palliative care teams at 388 core cancer treatment hospitals and 161 certified medical institutions that have palliative care units (PCUs).

**Results:**

Valid responses were obtained from 311 physicians (response rate, 56.7%) who were evenly distributed throughout the country without significant geographical biases. Kampo medicines were prescribed for controlling cancer-related symptoms by 64.3% of the physicians. The symptoms treated with Kampo medicines were numbness/hypoesthesia (n = 99, 49.5%), constipation (n = 76, 38.0%), anorexia/weight loss (n = 72, 36%), muscle cramps (n = 71, 35.5%) and languor/fatigue (n = 64, 32.0%). Regarding open issues about prescription, 60.7% (n = 173) of the physicians raised the issue that the dosage forms need to be better devised.

**Conclusions:**

To increase the clinical use of Kampo medicines, more evidence from clinical studies is necessary. In addition, their mechanisms of action should be clarified through laboratory studies.

## Background

### History of kampo medicine

Kampo medicines are traditional Japanese medicines produced from medicinal plants and herbs. Kampo originates from China and has been adapted to the Japanese culture [1]. Chinese herbal medicine was imported to Japan in 552 AD, after which it was uniquely developed into Japanese Kampo [2]. Traditional Chinese Medicine is deeply philosophical and ideological, while Japanese Kampo tends to be more practical and simplified, and relies little on Taoist or other Chinese philosophy [2].

Kampo medicines are currently of great interest to palliative care physicians because of their potential to alleviate the adverse side effects of cancer treatment and improve patients’ quality of life.

### Use of Kampo and CAM in Japan

In the past few decades, Kampo has reintegrated into modern medical practice, accompanied by a scientific reevaluation and critical examination of its relevance in conventional medicine [2,3]. Kampo has been used in addition or alternatively to conventional medicines [4]. Currently more than 70% of Japanese physicians prescribe Kampo medicines in daily clinical practices [5]. Previous survey research has reported that 76% of the general population in Japan and 50% of outpatients in Tokyo have used some form of CAM and that 10% of the general population and 19% of outpatients in Tokyo had used Kampo medicine prescribed by physicians within the last 12 months [6,7]. In addition, the prevalence of use of CAM by cancer patients was 44.6% in Japan [8]. Internationally, the estimates of CAM use are higher in East Asia and highest in Japan compared to the USA and European countries [9,10]. CAM is often used in palliative care settings where the goal is not cure but rather improvement in QOL [10].

To date, the Ministry of Health, Labour and Welfare (MHLW) has approved the use of 148 Kampo medicines, and the prescription of Kampo medicines is within the national health insurance system [3,11]. Although Kampo can be seen as orthodox from a historical Japanese perspective, it tends to be classified as Complementary and Alternative Medicine (CAM) according to Western conventions. The main reason for this is the lack of scientific evidence of its efficacy and the limited knowledge and spread of this therapy in other regions, especially outside of East Asia.

However, clinical studies of Kampo have been conducted in Japan, and its efficacy has been reported in research papers. For example, a randomized control trial demonstrated that the Kampo medicine *Rikkunshito* exerted greater effects in alleviating gastrointestinal symptoms than cisapride (a gastroprokinetic agent) [12]. The efficacy of *Rikkunshito* against non-ulcer dyspepsia (NUD) [13,14], gastrointestinal symptoms after gastrectomy (surgical NUD) [15], functional dyspepsia [16,17], and nausea and vomiting caused by selective serotonin reuptake inhibitors [18] has also been reported. Also, the Japanese Society for Oriental Medicine has compiled comprehensive data on randomized controlled trials of Kampo medicine in Japan, published as “Evidence Reports of Kampo Treatment” (EKAT) [19]. In addition to clinical trials, the potential mechanisms of action of Kampo medicines are also starting to be reported [20].

As described above, there is increasing evidence of the efficacy of Kampo medicines and increasing attention has been given to their clinical application. However, there has been no comprehensive investigation of the use of Kampo medicines in cancer treatment. Therefore, we conducted a nationwide survey of the current use of Kampo medicines for cancer-related treatment and of physicians’ attitudes toward using Kampo medicines in Japan.

## Methods

### Study sample and data collection

The survey was carried out between January and March of 2011, by mailing a self-administered anonymous questionnaire to 549 palliative care physicians who administer chemotherapy to cancer patients or who are involved in their terminal care. The palliative care teams in 388 core cancer treatment hospitals and 161 palliative care units (PCUs) within medical institutions were selected because they represent palliative care practice in Japan. This included all core cancer treatment hospitals and PCUs in Japan as of February 2011. Core cancer treatment hospitals are the medical facilities specified by the MHLW to provide high-quality expert care for cancer patients. These facilities are established within each prefecture in Japan, according to the principles set forth in the Cancer Control Act promulgated in April 2007. The contact information of subjects was obtained from a web site of the Cancer Control Information Center, National Cancer Center [21].

We did not specifically include general internists or surgeons who are not in charge of palliative care as subjects of the survey. This is because the certification system for the palliative care specialist is still immature in Japan and the attending physicians of palliative care teams and PCUs are often internists or surgeons.

### Questionnaire development

An eight-page, 18-item questionnaire was designed in Japanese. It covered four categories: (1) status of cancer treatment and use of Kampo medicines, (2) cancer cachexia and utilization of Kampo medicines (data not shown), (3) adverse side effects of anti-cancer drugs and utilization of Kampo medicines, and (4) background variables. Although the questionnaire was not formally validated, the questionnaire and its items were designed and formulated based upon the expert opinions of specialists from palliative care, medical oncology, Kampo medicine, and biological statistics, and also from literature reviews. It was finalized after testing several samples.

### Ethical considerations

We conducted this research in compliance with the Helsinki Declaration. We had requested an ethical review of this research from the ethical review committee of the National Cancer Center prior to commencement. However, since this research involves neither patients’ data nor intervention, the committee judged that this research should not be subjected to any Japanese medical research guidelines. Accordingly, the research was exempt from the requirement for formal ethical approval.

To ensure that informed consent was obtained, the questionnaire was sent to the physicians with a leaflet explaining the survey’s objectives and that (1) each subject was free to decide whether or not to answer the questions; (2) the collected data will be processed and analyzed anonymously; and (3) the data will be securely archived by the Research Secretariat. Consent was implied through the return of a completed questionnaire.

### Data analysis

The collected data were entered into an electronic database and analyzed using SPSS (IBM, New York, USA). Chi-squared tests (p value < 0.050) were conducted to compare the frequency distributions of two cross-tabulations. The first was physicians in the palliative care teams at the core cancer treatment hospitals compared with physicians in the PCUs. The second was the palliative care specialists certified by the Japan Society of Palliative Medicine (JSPM) compared with non-specialists.

## Results and discussion

Of the 549 questionnaires distributed, 311 valid responses were collected for analysis (response rate, 56.7%). Responses were obtained from 226 physicians (response rate, 58.2%) at core cancer treatment hospitals (palliative care team physicians) and 79 physicians (response rate, 49.1%) from PCUs (PCU physicians). With the moderate rate of valid responses (56.7%), the respondents were well-distributed throughout the country, without significant geographical biases. Table 1 shows the response rates and the respondents’ background characteristics. Two hundred thirty seven respondents (77.9%) were aged between 40 and 59 years. Two hundred seventy three respondents (90.1%) were male, and 128 respondents (41.2%) were JSPM-authorized palliative care specialists (including provisional medical advisors).

**Table 1:** Respondents’ background characteristics

**Respondents (n = 311)**			**Average ± SD**	**Minimum value**	**Maximum value**		
Age			49 ± 8	28	75		
Years of experience			23 ± 8	4	50		
			Responses	%			
Institution (n = 549) *							
Core cancer treatment hospital (n = 388)			226	58.2			
Palliative Care Unit in medical institution (n = 161)			79	49.1			
			n	%			
Age group							
20–29 years			1	0.3			
30–39 years			39	12.8			
40–49 years			119	39.1			
50–59 years			118	38.8			
≥ 60 years			27	8.9			
Sex							
Male			273	90.1			
Female			30	9.9			
Palliative Care Specialists certified by JSPM**							
Specialists (including provisional medical advisors)			128	41.2			
Non-specialists			183	58.8			
Region***	Hokkaido–Tohoku	Kanto	Chubu	Kinki	Chugoku	Shikoku	Kyushu–Okinawa
Number of questionnaires distributed	79	116	92	91	47	27	97
Number of responses	26	43	41	33	25	11	37
Response rate (%)	32.9	37.1	44.6	36.3	53.2	40.7	38.1

*Six responses had missing institution data, and ***95 responses had missing region data.

** JSPM: Japan Society for Palliative Medicine.

### Difficult to treat cancer-related symptoms

Physicians were asked to identify which of the 23 common cancer-related symptoms that they find difficult to treat (Table 2). More than 50% of the physicians identified *numbness/hypoesthesia* (n = 240, 77.2%), *languor/fatigue* (n = 225, 72.3%), *delirium* (N = 170, 54.7%), and *taste alteration* (n = 166, 53.4%). In comparison with the PCU physicians, more palliative care team physicians identified *taste alteration* (p = 0.029), *nausea/vomiting (during chemotherapy)* (p = 0.000), and *constipation (caused by opioid use)* (p = 0.038). More of the PCU physicians, on the other hand, reported having difficulty treating *adjustment disorder* (p = 0.014). In addition, the symptoms of *taste alteration* (p = 0.050), *dysphagia/deglutition disorder* (p = 0.036) and *muscle weakness* (p = 0.047) were identified as being difficult to treat more often by the palliative care specialists than the non-specialists.

**Table 2:** Difficult to treat cancer-related symptoms identified by physicians

**Symptoms**	**All physicians (n = 311)**		**Palliative care teams (n = 226)**		**PCUs (n = 79)**		**p-value**	**Specialists (n = 128)**		**Non-specialists (n = 183)**		**p-value**
	**frequency**	**%**	**frequency**	**%**	**frequency**	**%**		**frequency**	**%**	**frequency**	**%**	
Numbness/Hypesthesia	240	77.2	180	79.6	55	69.6	0.165	99	77.3	141	77.0	1.000
Languor/Fatigue	225	72.3	161	71.2	61	77.2	0.276	99	77.3	126	68.9	0.122
Delirium	170	54.7	119	52.7	48	60.8	0.447	73	57.0	97	53.0	0.490
Taste alteration	166	53.4	124	54.9	42	53.2	0.029	77	60.2	89	48.6	0.050
Edema (Local edema/Anasarca)	150	48.2	109	48.2	39	49.4	0.821	59	46.1	91	49.7	0.565
Pain	146	46.9	113	50.0	31	39.2	0.226	55	43.0	91	49.7	0.250
Anorexia/Weight loss	140	45.0	109	48.2	30	38.0	0.108	64	50.0	76	41.5	0.165
Abdominal discomfort	131	42.1	98	43.4	31	39.2	0.735	55	43.0	76	41.5	0.816
Stomatitis/Xerostomia	122	39.2	89	39.4	33	41.8	0.141	54	42.2	68	37.2	0.409
Depression	116	37.3	86	38.1	30	38.0	0.175	41	32.0	75	41.0	0.122
Adjustment disorder	113	36.3	73	32.3	39	49.4	0.014	47	36.7	66	36.1	1.000
Dyspnea/Breathlessness	113	36.3	77	34.1	35	44.3	0.162	48	37.5	65	35.5	0.811
Nausea/Vomiting (other)	101	32.5	75	33.2	24	30.4	0.893	38	29.7	63	34.4	0.392
Dysphagia/Deglutition disorder	100	32.2	68	30.1	31	39.2	0.281	50	39.1	50	27.3	0.036
Sleep disorder/Insomnia	93	29.9	69	30.5	23	29.1	0.796	42	32.8	51	27.9	0.379
Constipation (caused by opioid use)	84	27.0	69	30.5	15	19.0	0.038	34	26.6	50	27.3	0.898
Nausea/Vomiting (during chemotherapy)	76	24.4	71	31.4	5	6.3	0.000	27	21.1	49	26.8	0.284
Muscle weakness	65	20.9	46	20.4	19	24.1	0.346	34	26.6	31	16.9	0.047
Nausea/Vomiting (caused by opioid use)	61	19.6	51	22.6	10	12.7	0.690	24	18.8	37	20.2	0.774
Constipation (not caused by opioid use)	59	19.0	47	20.8	11	13.9	0.377	28	21.9	31	16.9	0.305
Muscle cramp	42	13.5	31	13.7	11	13.9	0.741	23	18.0	19	10.4	0.064
Diarrhea	40	12.9	34	15.0	6	7.6	0.136	16	12.5	24	13.1	1.000
Anemia	29	9.3	24	10.6	5	6.3	0.344	16	12.5	13	7.1	0.177
Others	11	3.5	6	2.7	5	6.3	0.325	4	3.1	7	3.8	0.770

Multiple answers allowed, p-value based on Chi-square test.

Numbness is a neuropathic symptom that frequently occurs as an adverse side effect of chemotherapy. It has been reported to account for 58% of all neurological symptoms experienced by cancer patients [22]. Fatigue is the most common cancer symptom [23], and was reported by 66% of patients in a previous study [22]. The prevalence of delirium is 25–40% (85–88% in the terminal stage of cancer) [24-26], and the prevalence of taste alteration is 36–75% among patients receiving chemotherapy [27]. Thus, it was shown in the present survey that the symptoms palliative care physicians have difficulty managing in Japan are those frequently seen in cancer patients.

We also found that the palliative care team physicians confront *taste alteration* (p = 0.029), *nausea/vomiting during chemotherapy* (p = 0.000) and *constipation during opioid use* (0.038) more often than the PCU physicians (Table 2). These facts suggest that the palliative care teams are often in charge of patients receiving chemotherapy, while PCUs are more frequently dealing with psychiatric symptoms than the adverse side effects of chemotherapy.

### Prescription of Kampo medicines

Kampo medicines were being prescribed by 64.3% (n = 200) of the physicians to alleviate the cancer patients’ symptoms. Kampo medicines were prescribed to control *numbness/hypoesthesia* (n = 99, 49.5%), *constipation (not caused by opioid use)* (n = 76, 38%), *anorexia/weight loss* (n = 72, 36%), *muscle cramps* (n = 71, 35.5%), and *languor/fatigue* (n = 64, 32%) by more than 30% of the physicians (Table 3). The palliative care team physicians prescribed Kampo medicines for *numbness/hypoesthesia* (p = 0.000), *anorexia/weight loss* (p = 0.046), *pain* (p = 0.020), and *nausea/vomiting during chemotherapy* (p = 0.016), more frequently than the PCU physicians. This difference may arise because the palliative care teams more often examine patients who are under chemotherapy than the PCUs, and thus they pay more attention than the PCUs to the necessity of controlling the adverse side effects of chemotherapy. Also, PCU patients have more difficulty taking Kampo medicines than the general hospital patients under the palliative care teams. The frequency of prescribing Kampo medicines did not vary significantly across the symptoms between the palliative care specialists and non-specialists.

**Table 3:** Symptoms for which Kampo medicines were prescribed

**Symptoms**	**All physicians (n = 200)**		**Palliative care teams (n = 149)**		**PCUs (n = 46)**		**p-value**
	**frequency**	**%**	**frequency**	**%**	**frequency**	**%**	
Numbness/Hypesthesia	99	49.5	86	57.7	12	26.1	0.000
Constipation (not caused by opioid use)	76	38	56	37.6	20	43.5	0.182
Anorexia/Weight loss	72	36	60	40.3	12	26.1	0.046
Muscle cramp	71	35.5	54	36.2	17	37.0	0.279
Languor/Fatigue	64	32	49	32.9	14	30.4	0.818
Constipation (caused by opioid use)	48	24	37	24.8	11	23.9	0.490
Abdominal discomfort	46	23	29	19.5	16	34.8	0.088
Diarrhea	45	22.5	39	26.2	5	10.9	0.090
Delirium	40	20	27	18.1	13	28.3	0.155
Pain	38	19	35	23.5	3	6.5	0.020
Edema (Local edema/Anasarca)	31	15.5	25	16.8	6	13.0	0.546
Nausea/Vomiting (other)	27	13.5	22	14.8	5	10.9	0.566
Nausea/Vomiting (during chemotherapy)	22	11	22	14.8	0	0.0	0.016
Stomatitis/Xerostomia	21	10.5	19	12.8	2	4.3	0.216
Taste alteration	20	10	17	11.4	3	6.5	0.409
Depression	20	10	17	11.4	3	6.5	0.409
Nausea/Vomiting (caused by opioid use)	17	8.5	16	10.7	1	2.2	0.129
Adjustment disorder	15	7.5	12	8.1	3	6.5	0.846
Sleep disorder/Insomnia	14	7	10	6.7	4	8.7	0.823
Others	13	6.5	6	4.0	6	13.0	0.055
Anemia	11	5.5	9	6.0	2	4.3	0.805
Dysphagia/Deglutition disorder	10	5	9	6.0	1	2.2	0.581
Dyspnea/Breathlessness	6	3	5	3.4	1	2.2	1.000
Muscle weakness	3	1.5	3	2.0	0	0.0	0.614

Multiple answers allowed, p-value based on Chai-square test.

### Reasons for prescription

More than 60% of the physicians prescribed Kampo medicines for the following reasons: ‘the drug therapy options are greater’ (n = 144, 72%), ‘ineffectiveness of other treatments’ (n = 129, 64.5%), and ‘unavailability of other appropriate treatments’ (n = 127, 63.5%). Although ‘patient demand’ was the least frequent reason (n = 46, 23%), palliative care specialists were more attentive to patients’ demands than non-specialists (n = 28, 37.3%, p = 0.000).

### Variety and frequency of prescriptions

Eight Kampo medicines were selected from the literature reviews to investigate frequency of prescription. Table 4 shows the composition of each Kampo medicine [28-30].*Daikenchuto* was the most frequently prescribed (n = 140, 70%) among eight major Kampo medicines (Table 5). This is probably because the efficacy of *Daikenchuto* for the treatment of gastrointestinal symptoms is currently being tested in clinical trials in Japan and the United States. A tolerability and efficacy phase II study of *Daikenchuto* for the treatment of postoperative ileus has been already completed in the United States [31]. This might encourage its prescription by physicians. The palliative care team physicans prescribed *Goshajinkigan* (p = 0.000)*, Rikkunshito* (p = 0.001)*, Hochuekkito* (p = 0.011)*, Juzentaihoto* (p = 0.001), and *Hangeshashinto* (p = 0.000) more frequently than PCU physicians, while there were no significant differences in the medicines prescribed between the palliative care specialists and non-specialists.

**Table 4:** Composition of Kampo medicines

**Kampo Medicine**	**Ingredients (crude drugs)**									
Hangeshashinto	Pinelliae Tuber	Scutellariae Radix	Zingiberis Processum Rhizoma	Glycyrrhizae Radix	Zizyphi Fructus	Ginseng Radix	Coptidis Rhizoma			
Hochuekkito	Astragali Radix	Atractylodis lanceae Rhizoma	Ginseng Radix	Angelicae Radix	Bupleuri Radix	Zizyphi Fructus	Aurantii Nobilis Pericarpium	Glycyrrhizae Radix	Cimicifugae Rhizoma	Zingiberis Rhizoma
Rikkunshito	Atractylodis lanceae Rhizoma	Ginseng Radix	Pinelliae Tuber	Poria	Zizyphi Fructus	Aurantii Nobilis Pericarpium	Glycyrrhizae Radix	Zingiberis Rhizoma		
Juzentaihoto	Astragali Radix	Cinnamomi Cortex	Rehmanniae Radix	Paeoniae Radix	Cnidii Rhizoma	Atractylodis lanceae Rhizoma	Angelicae Radix	Ginseng Radix	Poria	Glycyrrhizae Radix
Yokukansan	Atractylodis lanceae Rhizoma	Poria	Cnidii Rhizoma	Uncariae Uncis cum Ramulus	Angelicae Radix	Bupleuri Radix	Glycyrrhizae Radix			
Shakuyakukanzoto	Glycyrrhizae Radix	Paeoniae Radix								
Daikenchuto	Zingiberis Processum Rhizoma	Ginseng Radix	Zanthoxyli Fructus							
Goshajinkigan	Rehmanniae Radix	Achyranthis Radix	Corni Fructus	Dioscoreae Rhizoma	Plantaginis Semen	Alismatis Rhizoma	Poria	Moutan Cortex	Cinnamomi Cortex	Processi Aconiti Radix

Ingredients of each Kampo medicine were based on the package inserts of Tsumura products [28].

Scientific names of ingredients were based on Metabolomics.jp [29] and The Japanese Pharmacopeia Fifteenth edition [30].

**Table 5:** The Kampo medicines prescribed by the physicians

**Kampo medicine**	**All physicians (n = 200)**		**Palliative care teams (n = 149)**		**PCUs (n = 46)**		**p-value**
	**frequency**	**%**	**frequency**	**%**	**frequency**	**%**	
Daikenchuto	140	70.0	109	73.2	29	63.0	0.124
Goshajinkigan	100	50.0	89	59.7	11	23.9	0.000
Rikkunshito	97	48.5	82	55.0	15	32.6	0.001
Shakuyakukanzoto	96	48.0	76	51.0	20	43.5	0.069
Hochuekkito	90	45.0	76	51.0	13	28.3	0.011
Juzentaihoto	84	42.0	73	49.0	11	23.9	0.001
Yokukansan	61	30.5	45	30.2	16	34.8	0.253
Hangeshashinto	54	27.0	51	34.2	3	6.5	0.000
Others	24	12.0	20	13.4	4	8.7	0.457

Multiple answers allowed, p-value based on Chi-square test.

### Physician-recognized effectiveness

We investigated the physician-recognized effectiveness of eight Kampo medicines. Two symptoms from each Kampo medicine’s package insert were listed and the physicians were asked to indicate whether they believed the medicine effectively treated them (Table 6). More than 50% of the physicians recognized the effectiveness of *Hangeshashinto* against *diarrhea caused by chemotherapy* (n = 31, 53.4%), of *Hochuekkito* and *Juzentaihoto* against *fatigue* (n = 54, 56.3% and n = 50, 56.8% respectively), of *Rikkunshito* against *anorexia* (n = 46, 50%), of *Yokukansan* against *delirium* (n = 38, 63.3%), of *Shakuyakukanzoto* against *leg cramps* (n = 79, 82.3%)*,* and of *Daikenchuto* against *ileus* (n = 101, 78.9%) and *opioid-caused constipation and abdominal pain* (n = 62, 53.9%). There was no significant difference in the medicines recognized as effective between the palliative care team and PCU physicians, while the palliative care specialists seemed to be more aware of the effectiveness of *Rikkunshito* against *nausea* than non-specialists (p = 0.012) (Table 6). These results suggest that there is consensus among palliative care physicians regarding the effectiveness of particular Kampo medicines against particular symptoms.

**Table 6:** Physician-recognized effectiveness of Kampo medicines

**Kampo medicine**	**Symptoms**	**Recognized as effective**						
		**All physicians**		**Specialists**		**Non-specialists**		**p-value**
		**frequency/total**	**%**	**frequency/total**	**%**	**frequency/total**	**%**	
Hangeshashinto	Diarrhea caused by chemotherapy	31/58	53.4	10/22	45.5	21/36	58.3	0.420
	Nausea	10/45	22.2	3/21	14.3	7/24	29.2	0.296
Hochuekkito	Anorexia	44/90	48.9	14/36	38.9	30/54	55.6	0.137
	Fatigue	54/96	56.3	19/39	48.7	35/57	61.4	0.295
Rikkunshito	Nausea	36/82	43.9	9/34	26.5	27/48	56.3	0.012
	Anorexia	46/92	50.0	18/40	45.0	28/52	53.8	0.528
Juzentaihoto	Fatigue	50/88	56.8	17/33	51.5	33/55	60.0	0.508
	AE caused by chemotherapy or radiotherapy	27/58	46.6	7/22	31.8	20/36	55.6	0.106
Yokukansan	Delirium	38/60	63.3	18/26	69.2	20/34	58.8	0.433
	Anxiety	15/50	30.0	6/23	26.1	9/27	33.3	0.758
Shakuyakukanzoto	Leg cramps	79/96	82.3	36/43	83.7	43/53	81.1	0.794
	Abdominal pain	20/57	35.1	11/25	44.0	9/32	28.1	0.268
Daikenchuto	Ileus	101/128	78.9	35/48	72.9	66/80	82.5	0.263
	Opioid-caused constipation and abdominal pain	62/115	53.9	22/47	46.8	40/68	58.8	0.254
Goshajinkigan	Numbness of hands and feet	47/107	43.9	18/39	46.2	29/68	42.6	0.840
	Nocturia	13/60	21.7	4/26	15.4	9/34	26.5	0.358

Multiple answers allowed, p-value based on Chi-square test.

### Prescription considerations

In the questionnaire, the physicians were asked, “What are the important considerations when selecting a Kampo medicine for prescription?”. More than 80% of the physicians recognized the importance of ‘symptom-alleviating effects (alleviation of adverse side effects) (n = 173, 93%)’, ‘alleviation of symptoms that reduce QOL in the terminal stage of cancer’ (n = 162, 87.6%), ‘low incidence of adverse side effects’ (n = 157, 84.9%) and ‘easy to combine with other drugs’ (n = 149, 80.5%). The palliative care specialists tended to place more importance than the non-specialists on ‘patient demand’ (p = 0.050).

### Open issues for prescription

The questionnaire also asked the physicians to identify any open issues regarding the prescription of Kampo medicines (Table 7), revealing that 60.7% (n = 173) of the physicians were concerned that the dose and dosage forms need to be better devised for simpler administration. Kampo medicines are commonly prepared in granule form or as decoctions, and their administration method is nauseating for some patients. This issue may be related to the observation that “patient demand” was chosen least frequently as the reason for prescription. In the clinical field of palliative care, Kampo medicines are often mixed in a jelly for patients who have dysphagia. For future prescriptions, the administration forms need to be better devised from an adherence perspective. The second most frequently identified issue was the lack of scientific evidence for their efficacy, with 38.2% (n = 109) of the physicians highlighting the absence of evidence from placebo-controlled trails. Watanabe *et al.*[3] recently reported a summary of 135 peer-reviewed Kampo trials published between 1988 and 2007. According to their report, 106 trials were RCTs, and only 22 were placebo-controlled trials. In two-thirds of the trials, the sample size was less than 100 patients, and only 35 trials were published in English and the rest were in Japanese. Watanabe *et al.*[3] concluded that the overall quality of the research was low.

**Table 7:** Open issues about prescribing Kampo medicine (n = 285)

**Issue**	**frequency**	**%**
The dose and dosage forms need to be better devised for simpler application	173	60.7
No evidence of efficacy from placebo-controlled studies	109	38.2
Action mechanism of Kampo medicine is not yet elucidated	97	34.0
No opportunity to learn about Kampo medicines	90	31.6
Relatively weak effect	79	27.7
Drug interaction is uncertain	66	23.2
Production of effect is slow	56	19.6
Others	25	8.8
There are no issues	12	4.2

Multiple answers allowed.

## Conclusions

We conducted a nationwide survey of 311 physicians working in palliative care teams at core cancer treatment hospitals and PCUs within medical facilities. Kampo medicines were prescribed by a high proportion (n = 200, 64.3%) of the palliative care physicians and were expected to provide valid means of controlling the cancer patients’ symptoms or the adverse side effects of chemotherapy. Palliative care physicians appear to be aware of the effectiveness of Kampo medicines. However, they prescribe Kampo medicines only to a limited extent because of the lack of evidence for their efficacy. Hence, we believe that the collection of more evidence from clinical studies is desirable in Japan.

## Abbreviations

MHLW: Ministry of health labour and welfare; CAM: Complementary and alternative medicine; PCUs: Palliative care units.

## Competing interests

The authors declare that they have no competing interests. The authors were free to interpret the data according to a strict scientific rationale.

## Authors’ contributions

YU conceived the study idea and SI and TY contributed to the study design and concept. YU distributed and collected the questionnaires. SI, TY and TM processed and analyzed the data. SI and TM wrote the initial manuscript. All authors interpreted the data and approved the final manuscript.

## Pre-publication history

The pre-publication history for this paper can be accessed here:

http://www.biomedcentral.com/1472-6882/12/222/prepub
